# Supplementary material for: The causal relationship between the human gut microbiota and pyogenic arthritis: a Mendelian randomization study
Source: Front Cell Infect Microbiol. 2024 Nov 26;14:1452480. doi: 10.3389/fcimb.2024.1452480 (PMC11629706; doi:10.3389/fcimb.2024.1452480)
Supplement: Supplementary file 1 [file DataSheet1.zip › mendelian test/ebi-a-GCST90027830.csv_forest.pdf]

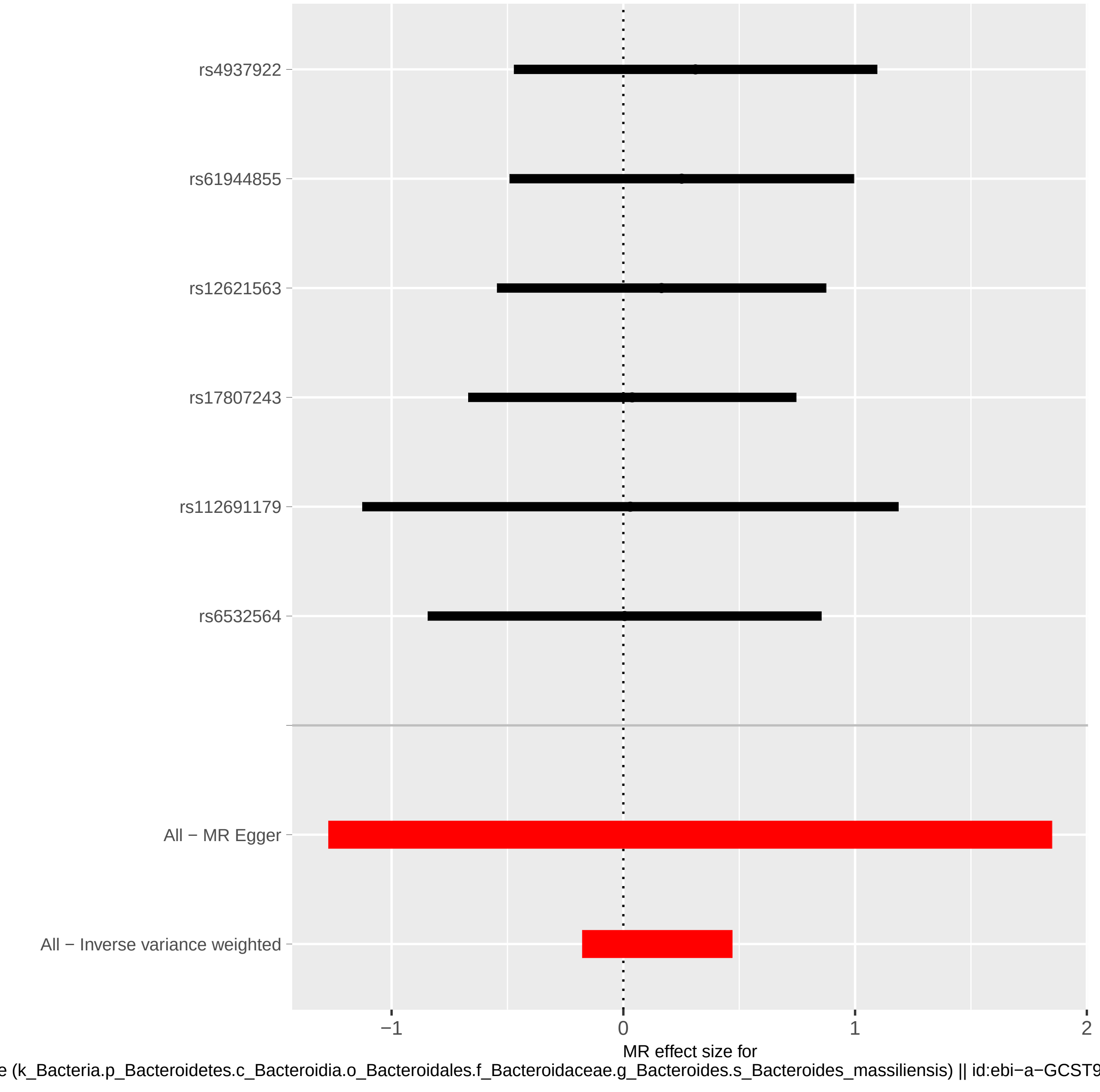
rs4937922
rs61944855
rs12621563
rs17807243
rs112691179
rs6532564
All − MR Egger
All − Inverse variance weighted
−1
0
1
2
MR effect size for
e (k_Bacteria.p_Bacteroidetes.c_Bacteroidia.o_Bacteroidales.f_Bacteroidaceae.g_Bacteroides.s_Bacteroides_massiliensis) || id:ebi−a−GCST9
